# Acute appendicitis due to *Cytomegalovirus* in an apparently immunocompetent patient: a case report

**DOI:** 10.1186/1752-1947-8-92

**Published:** 2014-03-10

**Authors:** Maria Bruna Pasticci, Simona Corsi, Francesca Spigarelli, Stefano Correnti, Daniela Francisci, Roberto Castronari, Pamela Baldin, Annapaola Prosperini, Franco Baldelli, Elio Cenci, Alessandra Sensini, Olivia Morelli

**Affiliations:** 1 Infectious Disease, Department Experimental Medicine and Biochemical Sciences, University of Perugia, 06100 Perugia, Italy; 2 Gastroenterology, Department of Clinical and Experimental Medicine, University of Perugia, 06100 Perugia, Italy; 3 Microbiology, Department Experimental Medicine and Biochemical Sciences, University of Perugia, 06100 Perugia, Italy; 4 Pathology and Diagnostic Cytology, Hospital Santa Maria della Misericordia, 06100 Perugia, Italy; 5 General Surgery, Department of Surgical Sciences, Hospital Santa Maria della Misericordia, 06100 Perugia, Italy; 6 Unit of Pathology, St’ Orsola Malpighi University Hospital, Bologna, Italy

**Keywords:** *Cytomegalovirus* appendicitis, Primary *Cytomegalovirus* infection, Primary sclerosing cholangitis, Ulcerative colitis

## Abstract

**Introduction:**

In healthy subjects, *Cytomegalovirus* infection can be asymptomatic or manifest as mononucleosis syndrome, but organ disease has also been reported. However, in immunocompromised patients this infection can lead to its most significant and severe disease and even mortality. When *Cytomegalovirus* causes a gastrointestinal tract infection, it more commonly manifests with luminal tract disease and is usually characterized by ulcerative lesions. Appendicitis is a rare manifestation, and has been reported mainly in human immunodeficiency virus-infected patients or patients with other causes of immunocompromise.

**Case presentation:**

The authors report on a case of acute primary *Cytomegalovirus* infection complicated with acute appendicitis due to *Cytomegalovirus* in an apparently immunocompetent 24-year-old Caucasian man also suffering from primary sclerosing cholangitis and ulcerative colitis. Diagnosis was based on clinical manifestations, serology results, as well as microbiological and histological findings. Treatment consisted of surgery and anti-*Cytomegalovirus* therapy.

**Conclusions:**

*Cytomegalovirus* should be included among the etiologic agents of acute appendicitis in patients with primary sclerosing cholangitis and ulcerative colitis. Currently, there are no definitive data regarding the frequency of *Cytomegalovirus* appendicitis and the role of anti-*Cytomegalovirus* treatment in human immunodeficiency virus-negative and apparently immunocompetent subjects.

## Introduction

In healthy subjects, *Cytomegalovirus* (CMV) infection can be asymptomatic or manifest as mononucleosis syndrome, but organ disease has also been observed [1-3]. However, in immunocompromised patients, CMV infection can lead to its most significant and severe disease manifestations. In bone marrow recipients, CMV pneumonia is the most common life-threatening infection after transplantation [1]. CMV infection is the most common viral infection in patients with human immunodeficiency virus (HIV) infection and CMV retinitis continues to be the most frequent sight-threatening infection in the era of highly active antiviral therapy [1]. In patients with HIV infection, CMV disease of the gastrointestinal tract is also frequently observed [1,2]. CMV infection of the gastrointestinal tract can occur anywhere in the gastrointestinal system but luminal tract disease is the most common localization and is usually characterized by ulcerative lesions. Esophagitis and colitis are the most frequent manifestations; however, CMV gastritis, small bowel enteritis, intestinal stricture, proctitis, cholangitis, hepatitis and pancreatitis have been observed [1-3]. CMV appendicitis is a rare manifestation, reported mainly in HIV infected patients [4,5]. Less frequently involved are patients with other causes of immunocompromise [6-8] and very few cases have been reported in apparently immunocompetent subjects [9-12]. However, Dzabic *et al*. have evidenced cells which were double positive for both early CMV antigens and interleukin (IL)-6 and/or IL-8 in 63% of patients with confirmed acute appendicitis and have found a possible correlation with CMV infection and the severity of disease [11]. CMV was also the most frequently detected virus in a study including 38 children who had undergone appendectomy for acute appendicitis [12].

The authors report a case of acute primary *Cytomegalovirus* infection complicated with acute appendicitis due to *Cytomegalovirus* in an apparently immunocompetent patient also suffering from primary sclerosing cholangitis (PSC) and ulcerative colitis (UC).

## Case presentation

**Figure 1:**
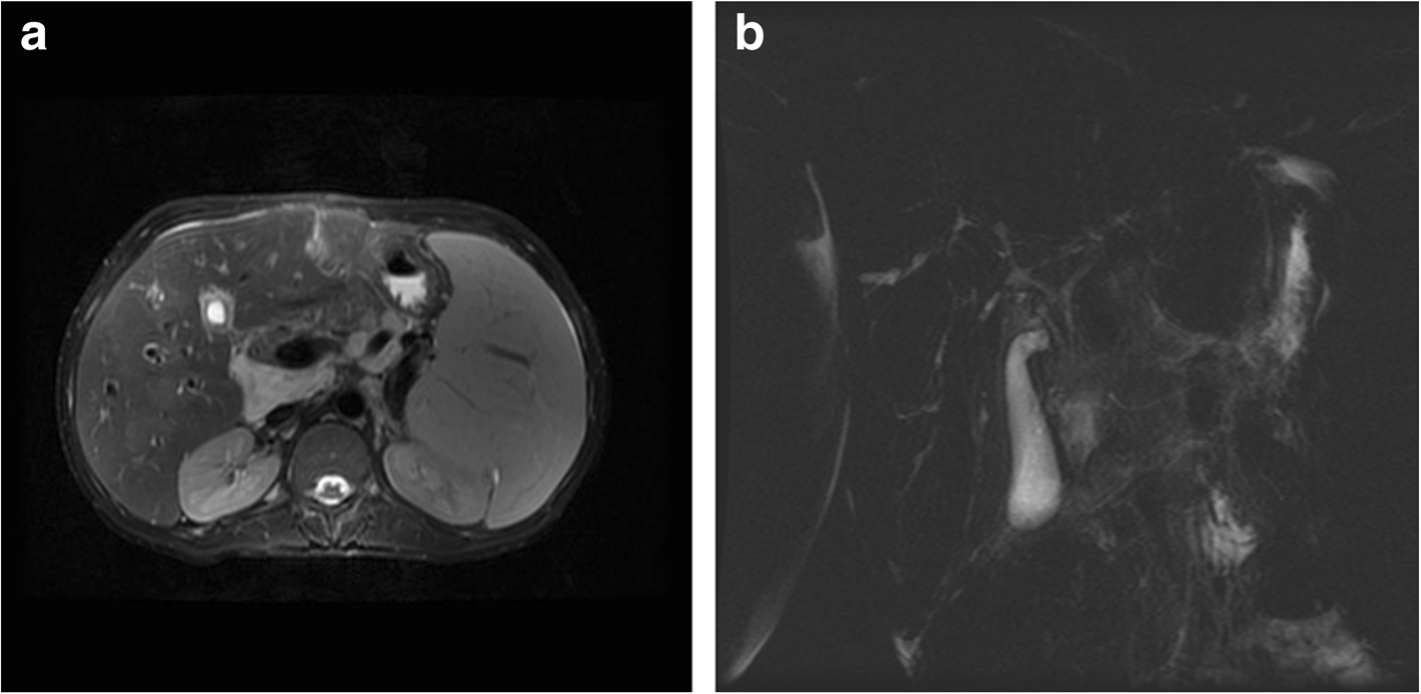
Magnetic resonance showing bile duct irregularities (a); T2-weighted image, depicting hepatic hilum tissue with contrast enhancement (b).

A 24-year-old Caucasian man with fever and upper quadrant abdominal pain over the previous 20 days was admitted to our hospital. Before admission, ciprofloxacin and metronidazole, followed by cefixime had been prescribed. Six years prior, the patient had been diagnosed with PSC, UC, suspected retroperitoneal fibrosis, bile sludge and splenomegaly. For this, he was prescribed ursodiol 300mg BID and mesalamine 4g per day. At that time, investigations included exploratory laparotomy and a biopsy of the perihepatic, retroperitoneal tissue which excluded malignancy. Over this six-year period, the patient presented with recurrent episodes of cholangitis, the serum level of aminotransferases remained substantially normal while there was a progressive worsening of cholestatic test results and a progressive liver enlargement along with fibrosis. Specifically, gamma glutamyl transpeptidase (GGT) and alkaline phosphatase (ALP) increased from 141UI/L to 344UI/L and 847UI/L to 2534UI/L, respectively. Hepatic tissue stiffness, measured by FibroScan®, progressed from 12.6 to 17.3kPa. The patient was never treated with immunosuppressive therapy or corticosteroids. One month before admission, an upper endoscopy was performed which excluded esophageal varices. One week before admission, a magnetic resonance of his abdomen and bile ducts revealed further enlargement of the liver, spleen and the tissue surrounding his hepatic hilum (Figure 1a), posterior to the pancreas head. The latter caused a compression of his second duodenal tract and a wrapping of the splenic and hepatic arteries. Beading and narrowing of the intra-hepatic and common bile ducts (Figure 1b) resulted in more extension and a narrowing of the pancreatic duct was also reported.

**Figure 2:**
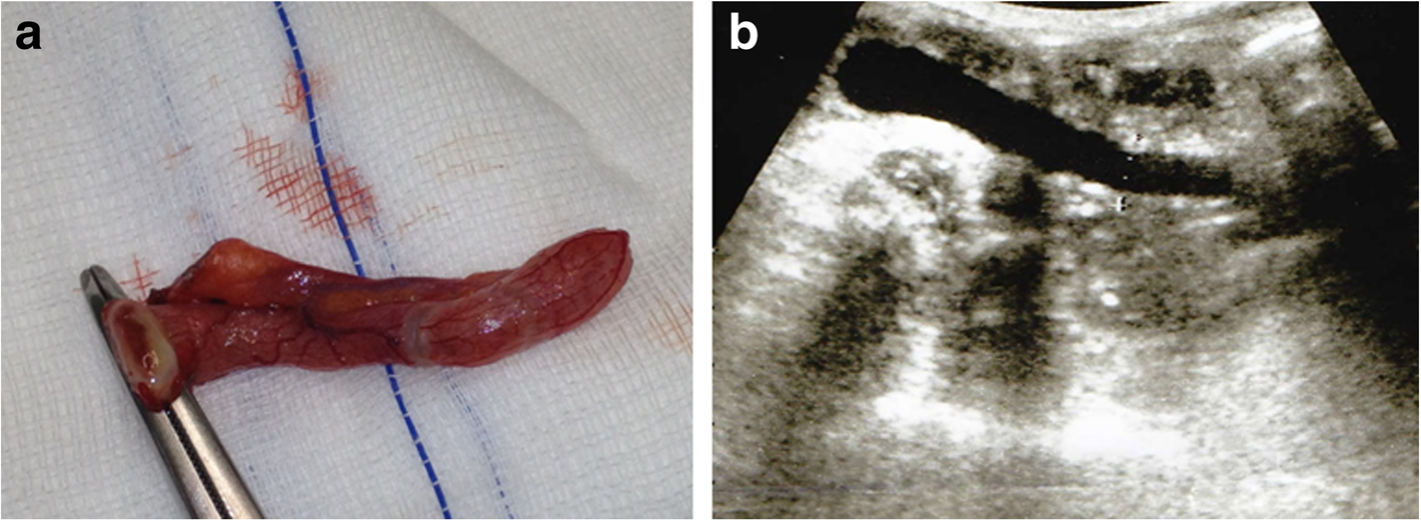
Acute catarrhal appendicitis (a); abdominal ultrasound showing “finger in glove” anechoic image with incompressible lumen (b).

**Figure 3:**
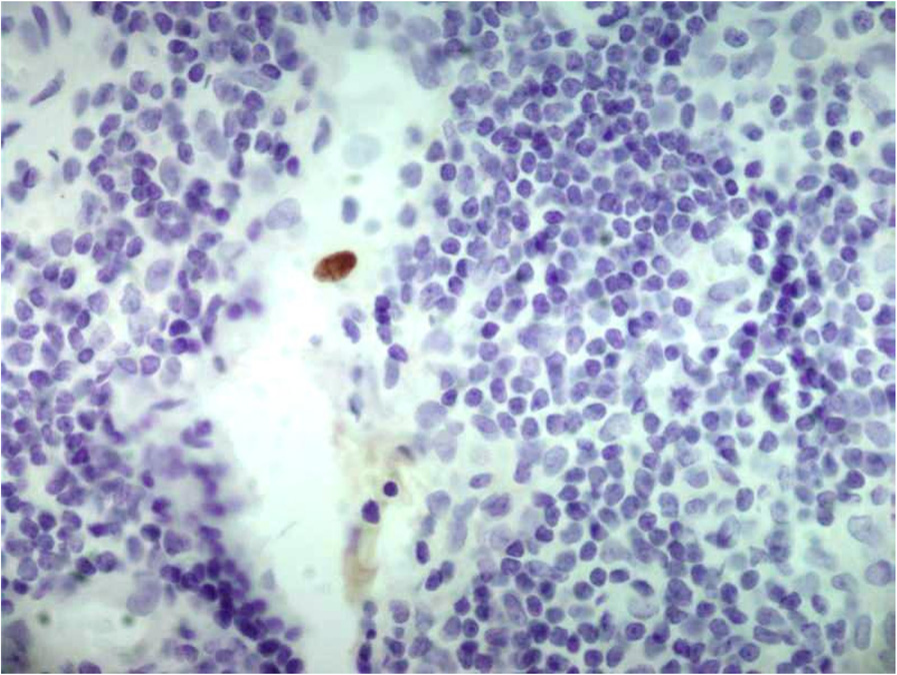
**Appendix section: early *Cytomegalovirus *** **antigens (Monoclonal Mouse Anti-Cytomegalovirus Clone CCH2?+?DDG9, Ventana Medical System, Roche, USA).**

At admission, our patient had a fever of 38.8°C and physical examination revealed tenderness of his epigastrium and right upper hypochondrium. Results from blood tests are reported in Table 1. Microbiological blood and urine investigations were negative for bacteria. A chest radiograph was normal while an abdominal sonography revealed an enlarged liver, thickened choledocus, dilatation of the intra-hepatic biliary tree, splenomegaly and lymphadenopathy of the hepatic hilus. A colonoscopy showed erythema of the colonic mucosa from the rectum to the cecum, with areas of increased erythema and telangiectasia in the ascending colon. Random biopsy showed focal atrophy of the colonic mucosa with edema and chronic inflammatory infiltrates, but specific investigations for CMV were not carried out. Imipenem 500mg IV four times daily was administered. Three days later, due to our patient’s persisting fever and abdominal pain, imipenem was substituted with tigecycline 50mg IV BID. Further blood and urine cultures for bacteria were negative. Both the erythrocyte sedimentation rate (ESR) and C-reactive protein level (C-RP) remained high, whereas the white blood cell (WBC) and neutrophil counts decreased (Table 1) and the procalcitonin level was 0.38ng/ml. The fever persisted while the upper abdominal pain subsided slightly. Investigations for HIV, *Toxoplasma gondii*, CMV, measles, parotitis and hepatitis C virus (HCV) all were negative, while results for varicella zoster virus, human herpes virus, Epstein Barr, rubella, and parvo virus B19 indicated previous infection. CD4?+?T lymphocytes were 1055mm^3^ (20.3%) and CD3?+?T lymphocytes were 3193mm^3^ (67%). *Mycobacterium tuberculosis* interferon gamma release assay (QuantiFERON®–TB Gold, Cellestis Limited, Carnegie, Victoria, Australia) showed negative results. Twelve days after admission, teicoplanin 400mg die, gentamicin 80mg TID, metronidazole 500mg TID were prescribed, while tigecycline was stopped. Two days later, deoxyribonucleic acid (DNA) *Cytomegalovirus* (Q-CMV Real Time, Nanogen Advanced Diagnostics, Torino, Italy) was detected in the blood with ≤253 copies/mL. Three days later, this value increased to 6189 copies/mL, while 1431 copies/mL were evidenced from a urine sample, the CMV pp65-antigen (Indirect Immunofluorescence, anti-CMV pp-UL83, Argene, France) was also positive, and CMV serology indicated acute CMV infection (Table 1). Our patient’s fever rose to 39.2°C and his abdominal pain extended to the right lower abdominal quadrant with radiation to the right groin and right testicle. Ultrasound (Figure 2a) suggested acute appendicitis and he underwent surgery (Figure 2b). Histology showed inflammatory infiltrates, including lymphocytes and neutrophils, while histochemistry was positive for CMV early antigens (Monoclonal Mouse Anti-Cytomegalovirus Clone CCH2?+?DDG9, Ventana Medical System, Roche, USA) (Figure 3). Real time reaction and shell vial culture of the appendix tissue also were positive. Microbiological investigations for bacteria and fungi showed *Peptococcus* spp. and *Candida albicans*. Teicoplanin, gentamicin and metronidazole were administered for a total of 12 days along with intravenous ganciclovir 5mg/kg twice for 15 days. After discharge, oral valganciclovir 900mg BID was prescribed for 10 days. After this, the CMV nucleic acid in the blood and urine was negative while ESR and cholestatic liver test results remained abnormally high (Table 1).

**Table 1:** Laboratory tests

	**2/22/2012**	**3/3/2012**	**3/6/2012**	**3/7/2012**	**3/19/2012**	**04/11/2012**
WBCs (3.60 to 9.60 × 10^3^μL)	14.45	8.19	9.02	8.6	9.43	8.7
Neutrophils (42.0 to 75.0%)	80.9	34.0	38.0	48	62	69.3
Lymphocytes (20.5 to 51.1%)	11.7	57.0	52.8	43	30	19
Monocytes (1.0 to 10.0%)	6.6	5.0	5.3	8	6	9
Eosinophils (≤5%)	0.5	0.0	0.7	0	2	2.2
Basophils (≤1%)	0.3	3.0	3.2	1	0	0.5
CD4?+?T (430 to 1590mm^3^, 30 to 70%)			1055 (20.3%)		1132 (41%)	
CD8?+ T (220 to 1040mm^3^, 13 to 40%)			3193 (67%)		849 (31%)	
Hb (13.0 to 17.0g/dL)	12.3	10.9	9.9	9.9	10.2	10.5
RBCs (4.30 to 5.80 × 10^6^μL)	4.26	3.93	3.54	3.58	3.55	3.74
I.N.R (0.80 to 1.2)	1.40				1.4	1.2
PLT (140 to 440 × 103μL)	374	215			383	355
Albumin (3.5 to 4.5g/dL)	4.2				3.9	
ALT (0 to 45UI/L)	56	35	36	20	16	65
AST (0 to 45UI/L)	66	65	70	34	36	58
GGT (7 to 49UI/L)	344	197	197	181	266	277
ALP (80 to 320UI/L)	2534	1819	1671	1360	1652	-
TB (0.00 to 1.20mg/dL)	1.91	1.2	1.69	1.2	1.27	1.44
DB (0.00 to 0.25mg/dL)	1.37	0.79	1.18	0.86	0.88	1.0
Amylase (30 to 118) UI/L	44					47
Immunoglobuline IgG (650 to 1600mg/dL)	3360					
IgG4 (110 to 1570mg/dL)	521					
Azotemia (10 to 50mg/dL)	22				14	
Creatinine (0.50 to 1.40mg/dL)	0.68				0.5	
ESR (1 to 25 1°h)	104	81	120	99	120	120
C-RP (0.0 to 0.5mg/dL)	4.8	2.8	7.7	5.4	1.6	
CMV-IgG (<20U/mL neg)	11.4		79			65
CMV-IgM (<20U/mL neg)	11.2		69			43
CMV-Avidity Index <0.5 low			0.2			0.1
Blood CMV-PCR (copies/mL)		<253	6189			
Urine CMV-PCR (copies/mL)			1431			
Appendix CMV-PCR *(copies/mL)				1210		
Appendix culture				Positive		
Appendix immunohistochemistry				Positive		

ALP, alkaline phosphatase; ALT, alanine aminotransferase; AST, aspartate aminotransferase; C-RP, C Reactive Protein; DB, direct bilirubin; ESR, erythrocyte sedimentation rate; GGT, gamma-glutamyl transpeptidase; I.N.R, international normalized ratio; PLT, platelets; RBCs, red blood cells; TB, total bilirubin; WBCs, white blood cells.

*DNA-CMV was quantified (Q-CMV Real Time, Nanogen Advanced Diagnostics, Torino, Italy) after tissue digestion for 30 minutes at 56°C with proteinase K buffer 500μL (Diatech Laboratories, Jesi, Italy) and DNA extraction with EasyMAG (bioMerieux, Merci L’Etoile, France), following the manufacturers’ instructions.

## Discussion

Primary sclerosing cholangitis is a disease of the bile ducts that causes inflammation and subsequent obstruction of the intra-hepatic and extra-hepatic bile ducts. Over time, the inflamed ducts develop scar tissue and this disrupts bile flow [13]. In addition to bile duct disease, 60 to 80% of patients with PSC have inflammatory bowel disease, typically ulcerative colitis [13]. Patients with PSC-UC may have a different phenotype compared to classic UC. Despite minimal endoscopic activity, these patients have more extensive colon involvement, more active histology inflammation and an increased risk of colorectal cancer [13,14]. An inverse prognostic relationship between PSC and UC has also been observed and progressive PSC requiring a liver transplant seems to be associated with UC, that is less symptomatic and less often requires colectomy [13]. Florin *et al*. investigated for an interaction between appendectomy and PSC in the epidemiology and clinical behavior of PSC-UC, finding no lower rates of appendectomy in PSC patients. However, prior appendectomy appeared to be associated with approximately a five-year delay in the onset of intestinal or hepatic disease [15]. A subgroup of patients with PSC having an overlap syndrome characterized by lymphoplasmacytic infiltrates, rich in IgG4-positive cells, has been identified [13,16]. Similar to classic PSC, these patients may have other autoimmune disorders, including autoimmune pancreatitis, autoimmune hepatitis, inflammatory bowel diseases, Sjögren's syndrome, nephritis and retroperitoneal fibrosis [13,16,17]. This immunoglobulin overlapping syndrome has been reported to be ameliorated with corticosteroid therapy [13,16]. Regarding our case report, the normal value of IgG4 excluded a diagnosis of PSC with overlapping IgG4 disease [13,16]. Moreover, the retroperitoneal fibrosis had a mild clinical progressive behavior. For these reasons, our patient was never treated with corticosteriods or immunosuppressive therapy.

CMV has also been implicated as a possible etiology in sclerosing cholangitis-like syndrome in patients with HIV infection [1,18]. CMV infection was not the cause of liver and bile duct disease in our patient. Also, clinical laboratory and endoscopic findings did not indicate CMV colitis [1,2,19]. In fact, typical CMV endoscopic findings were not detected, while CMV antibodies and CMV antigens were positive only 10 days after our patient was admitted.

At admission, our patient presented with right upper abdominal pain, elevated leucocyte and neutrophil counts, increased bilirubin, GGT and ALP levels. Therefore, recurrent bacterial cholangitis was the admitting diagnosis. However, despite an initial improvement with the administration of antimicrobials, his condition worsened due to increased fever and abdominal pain, also involving, at that time, the right lower abdominal quadrant, right groin and testicle. Acute appendicitis was diagnosed based on the clinical and ultrasound findings. Simultaneously, laboratory results indicated acute CMV infection; acute appendicitis due to CMV complicating acute CMV infection was suspected. Anti-CMV treatment was added to antimicrobials and our patient underwent surgery.

In our patient, CMV acute infection and acute CMV appendicitis were diagnosed based on: 1) CMV serology; 2) CMV DNA in the blood; 3) peripheral blood lymphocytosis; 4) the presence of CMV early antigen with immunohistochemistry in the appendix; 5) evidence from the literature that CMV gastrointestinal diseases, including florid appendicitis, can also occur in patients apparently non-immunocompromised [2,3,8-12]. Our patient was HIV negative and was never treated with immunosuppressive drugs; however, it is plausible that the chronic inflammatory state involving the intra-hepatic and common bile ducts and the colon or the primary CMV infection itself induced a temporary moderate lowering of CD4+ T lymphocytes causing a change in the immune reactivity favoring CMV organ disease [2,3,20]. The absence of intra-nuclear “owl’s eye” in the histology cannot exclude the diagnosis, given that this specific histological finding has a lower sensitivity than immunohistochemistry and molecular diagnostic methods [21].

## Conclusions

CMV should be included among the etiologic agents of acute appendicitis in patients with primary sclerosing cholangitis and ulcerative colitis. Currently, there are no definitive data regarding the frequency of CMV appendicitis and the role of anti-CMV treatment in HIV negative and apparently immunocompetent subjects.

## Consent

Written informed consent was obtained from the patient for publication of this case report and any accompanying images. A copy of the written consent is available for review by the Editor-in-Chief of this journal.

## Abbreviations

ALP: Alkaline phosphatase; ALT: Alanine aminotransferase; AST: Aspartate aminotransferase; BID: Two times a day; CMV: *Cytomegalovirus*; CMV-IgM: CMV immunoglobulin M; CMV-PCR: CMV-polymerase chain reaction; C-RP: C-reactive protein; DB: Direct bilirubin; ESR: Erythrocyte sedimentation rate; GGT: Gamma glutamyl transpeptidase; HCV: Hepatitis C virus; HIV: Human immunodeficiency virus; IgG: Immunoglobulin G; IL-6: Interleukin-6; IL-8: Interleukin-8; I.N.R: International normalized ratio; IV: Intravenous; kPa: Kilopascal pressure; PLT: Platelets; PSC: Primary sclerosing cholangitis; RBC: Red blood cell; TB: Total bilirubin; TID: Three times a day; UC: Ulcerative colitis; WBC: White blood cell.

## Competing interests

The authors declare that they have no competing interests.

## Authors’ contributions

PMB, SC, FS and OM cared for our patient, acquired, analyzed and interpreted the patient’s data, reviewed the literature, and were the major contributors in writing the manuscript. SC was involved with our patient’s care and reviewed the manuscript for important intellectual content. DF, RC, EC and AS carried out microbiological testing and reviewed the manuscript for important intellectual content. PB and AP did histological testing and reviewed the manuscript for important intellectual content. FB reviewed the manuscript and gave the final approval of the version to be published. All authors read and approved the final manuscript.
